# Sequence periodicity in nucleosomal DNA and intrinsic curvature

**DOI:** 10.1186/1472-6807-10-S1-S8

**Published:** 2010-05-17

**Authors:** T Murlidharan Nair

**Affiliations:** 1 Department of Biological sciences, Indiana University South Bend, 1700 Mishawaka Ave, South Bend, IN-46634, USA; 2 Department of Computer science/Informatics, Indiana University South Bend, 1700 Mishawaka Ave, South Bend, IN-46634, USA

## Abstract

**Background:**

Most eukaryotic DNA contained in the nucleus is packaged by wrapping DNA around histone octamers. Histones are ubiquitous and bind most regions of chromosomal DNA. In order to achieve smooth wrapping of the DNA around the histone octamer, the DNA duplex should be able to deform and should possess intrinsic curvature. The deformability of DNA is a result of the non-parallelness of base pair stacks. The stacking interaction between base pairs is sequence dependent. The higher the stacking energy the more rigid the DNA helix, thus it is natural to expect that sequences that are involved in wrapping around the histone octamer should be unstacked and possess intrinsic curvature. Intrinsic curvature has been shown to be dictated by the periodic recurrence of certain dinucleotides. Several genome-wide studies directed towards mapping of nucleosome positions have revealed periodicity associated with certain stretches of sequences. In the current study, these sequences have been analyzed with a view to understand their sequence-dependent structures.

**Results:**

Higher order DNA structures and the distribution of molecular bend loci associated with 146 base nucleosome core DNA sequence from* C. elegans* and chicken have been analyzed using the theoretical model for DNA curvature. The curvature dispersion calculated by cyclically permuting the sequences revealed that the molecular bend loci were delocalized throughout the nucleosome core region and had varying degrees of intrinsic curvature.

**Conclusions:**

The higher order structures associated with nucleosomes of* C.elegans* and chicken calculated from the sequences revealed heterogeneity with respect to the deviation of the DNA axis. The results points to the possibility of context dependent curvature of varying degrees to be associated with nucleosomal DNA.

## Background

Eukaryotic chromosome consists of a single DNA molecule that has been compacted several thousand fold by interacting with highly conserved proteins called core histones. The complex so formed is called the chromatin. The basic structural repeating unit of eukaryotic chromatin is the nucleosome [1-3]. The nucleosome core is made up of 146 bp of negatively charged DNA wrapped 1.65 times around highly basic proteins called histones, which neutralizes the negative charge. The histone proteins H2A, H2B, H3 and H4 make up the histone core. The formation of the chromatin facilitates the packaging of DNA into chromosomes by compacting it several thousand folds. While compaction facilitates easy packaging of DNA, it hinders the macromolecular machinery from reading the genetic code. Chemical and compositional modification of nucleosomes and nucleosome positioning plays an important role in gene regulation. For long it was thought that histones bound DNA randomly and were simply assigned the role of packaging proteins. Recent studies have thrown light into the basic organization of nucleosomes on chromosomes and their role in regulating genomic function (reviewed in [4]).

New technologies have paved the way towards genome-wide mapping of nucleosome positions, and several maps have now been published [5-9]. On one hand, as nucleosomes are ubiquitous in the chromosome, it has been debated whether there is a chromatin code, and that the nucleosome position might be regulated by the cell [10,11]. While on the other hand, analysis of sequence data from genome-wide maps have been used in understanding nucleosome organization and the underlying hidden signals for nucleosome positioning [12-15]. Periodicity of dinucleotides in chromatin was first noted by Trifonov [16-18]. There have been several reports delineating periodicity and sequence patterns associated with nucleosomal DNA since then [4,6,19]. Briefly, some of the signals that could potentially play a role in nucleosome positioning include signals for rotational and/or translational positioning [13]. The signals may be specific or degenerate, periodically dispersed or localized. In the dispersed category there are short stretches of sequences whose effects are magnified because of their repetitive appearance in a periodical manner [20]. There have been two schools of thought to explain the nucleosome code, viz. the counter-phase school and the in-phase school. According to the counter-phase school, the RR and YY dinucleotides dispersed along the nucleosome (where R=G or A, and Y=C or T), are not in the same phase when they repeat (i.e. they are in alternating RR/YY pattern) [16,21,22]. The in-phase school argues that RR and YY dinucleotides are in the same phase when they repeat [14,23-25]. While it is important to understand the nucleosomal DNA signals in terms of the sequence patterns embedded in them, it is equally important to understand the structures that these repeats impart to the free nucleosome DNA. Recent reports have revealed the bendability sequence pattern associated with nucleosome DNA [18] and report the sequence CCGGRATTYCCGG as the theoretically predicted common pattern of DNA bendability in the nucleosome. The pattern has been derived based on bendability properties that take into account the periodic occurrence of dinucleotides [26]. Unstacking of dinucleotides is a major contributor of DNA deformability/bendability [27]. Further, it is now well established that the structure of DNA is a function of its sequence [16,28-31] and certain short stretches of sequences have preference for a specific DNA structure. For instance, occurrence of AA/TT is known to intrinsically curve the DNA axis, while (CA)n or (CG)n form Z-DNA structures [13].Since DNA has to wrap around the histone octamer for nucleosome formation, having sequences that have the ability to naturally curve would facilitate the wrapping process. Curved DNAs have thus been considered as signals that could be involved in nucleosome positioning [32,33]. Recent reports have revealed a periodicity of AA and TT dinucleotides at an interval of 10.4 bp within the nucleosomes which could also potentially contribute to DNA curvature [26,34,35]. There is also a good agreement between the intrinsically curved DNA and model based prediction of nucleosome positioning [36].

Intrinsically curved DNA has been extensively investigated experimentally and theoretically [28,37-40]. Two classes of models have been proposed to explain the sequence-dependent structure of DNA. The wedge model which is based on the assumption that the hypothetical wedges that are formed as a result of non-coplanar base planes, when repeated in phase with DNA helix repeat (10.5 bp) produces macroscopic curvature [16,17,40]. The junction bending model attributes DNA curvature to the distortions at the junction between different DNA structural forms [29,41,42]. Both models agree that the overall curvature is additive over the individual bending elements and require the phasing of (A)n tracts. DNA curvature has also been demonstrated in DNA fragments lacking poly-A tracts [43]. Experimentally, DNA curvature is detected by the anomalous reptation of curved DNA during polyacrylamide gel electrophoresis [29,38]. Mobility of DNA in gel is directly related to the mean square end to end distance [44]. Wu and Crothers have designed an elegant gel electrophoretic permutation assay to localize the bending locus of an intrinsically curved DNA fragment [45]. De Santis* et al.*[46-48] have proposed a theoretical model for DNA curvature, and have shown that curvature dispersion is linearly correlated with gel electrophoretic retardation. The model has been experimentally verified and has been applied to analyze several systems [38,49].

In the present study, higher order DNA structures associated with 146 base nucleosome core DNA sequence from* C. elegans *[9] and nucleosomes from chicken [23] have been analyzed theoretically. Curvature dispersion associated with the 146 base nucleosome core DNA sequence has been calculated by cyclically permuting the sequence and the distribution of the molecular bend locus of the nucleosome core regions determined. The results indicate a wide distribution of the bend locus, having delocalized curvature throughout the nucleosome core region.

## Methods

### Data

The data for the current study were taken from the* C. elegans* UUPc (Unique unambiguous pyrocore) database [9] and collection of 177 natural nucleosomes from chicken [23] (Travers personal communication). The UUPc database contained 28,230 sequences from chromosome I, 30,310 sequences from chromosome II, 26,111 sequences from chromosome III, 30,177 sequences from chromosome IV, 39,547 sequences from chromosome V, and 33,488 sequences from chromosome X [9]. Both of the data sets had revealed ~10 bp periodicity with respect to AA/TT/TA dinucleotides.

### Curvature dispersion calculation

Curvature dispersion has been calculated following the model proposed by De Santis *et al*. [46]. The model uses conformational energy calculations to approximate the local deviations of the 16 different dinucleotide steps from the standard B-DNA structure. Deviations from the canonical B-DNA structure are integrated and represented as a curvature vector* C*(*n*, *v*), which represents the directional change of the double helical axis between sequence number* n* and *n + v.* Curvature vector per turn of B-DNA is given by:

Where* v^°^* is the average periodicity of DNA (10.4) and *d_j_ = r_j_ – i τ_j_*. Here* r*  and *τ* are the roll and tilt angles for different base pair dinucleotide fragment of DNA. The dispersion of curvature σ^2^ is calculated as the second moment of the curvature vector* C *(*n*, *v*) and is shown to be linearly correlated with electrophoretic retardation [47]. Calculating σ^2^ by cyclically permuting the sequence is a theoretical alternative for localizing the molecular bend locus. For details refer to De Santis et al. [46].

### DNA path calculation

DNA path was calculated using the model developed by Shpigelman *et al.*[50]. The overall DNA path is calculated using the local helix parameters viz. helix twist angle, wedge angle and the direction of deflection angle. The coordinates of the successive base pair stacks are calculated by applying (i) translation by half the average rise per residue (average rise per residue =3.39Å) along the Z axis (ii) half the helical twist rotation about Z-axis (iii) rotation by the wedge angle in the XY-plane, (iv) rotation by another half helical twist about the Z-axis and (v) translation by another half of the average rise per residue. These transformations can be described in the following equation

*where*

*and R_n_ is the rotation about n axis*

The programs for computing the coordinates were developed in R (http://www.r-project.org). The angles of Twist (Ω), Wedge (σ) and Direction (δ) were taken from those determined by Bolshoy *et al. *[43,50] experimentally as well as those determined by De Santis* et al. *[46,47,51]. Both set of angles essentially predicted the same structures. 

## Results and discussion

DNA sequences vary in their ability to deform and this is a direct function of their sequence. The variable deformability has a direct impact on how the DNA fragment wraps around the histone octamer. Approximately a 10-11 bp periodic recurrence of certain dinucleotides (AA/TT/TA) have been demonstrated in nucleosomal DNA. With a view to understand how this periodicity of certain dinucleotides translates into intrinsic deformability, the sequence-directed structures associated with the nucleosome DNA of* C. elegans* and chicken nucleosome have been analyzed using the theoretical models for DNA curvature. Recent analysis by measuring the distance between YY, YR, RR and RY dinucleotides of nucleosome DNA fragments from* C. elegans* revealed a consensus sequence structure of the nucleosome DNA repeat to be (YYYYYRRRRR)n [20]. Phase shifts between various dinucleotides within ~10 base nucleosome sequence repeat have been reported earlier [26,52]. A bendability matrix has been used to represent these phase preferences, and it has been noted that AA and TT dinucleotides counter-phase one another and may reflect the periodical pattern of the nucleosome DNA [26]. Nucleosome DNA bendability matrix that was recently determined from nucleosome core DNA sequences of* C. elegans* revealed a consensus repeat of A(TTTCCGGAAA)T [53]. To understand how the periodicity affects the overall structure of free nucleosomal DNA, the UUPc database and chicken nucleosome DNA were analyzed using the theoretical models for DNA curvature.

**Figure 1:**
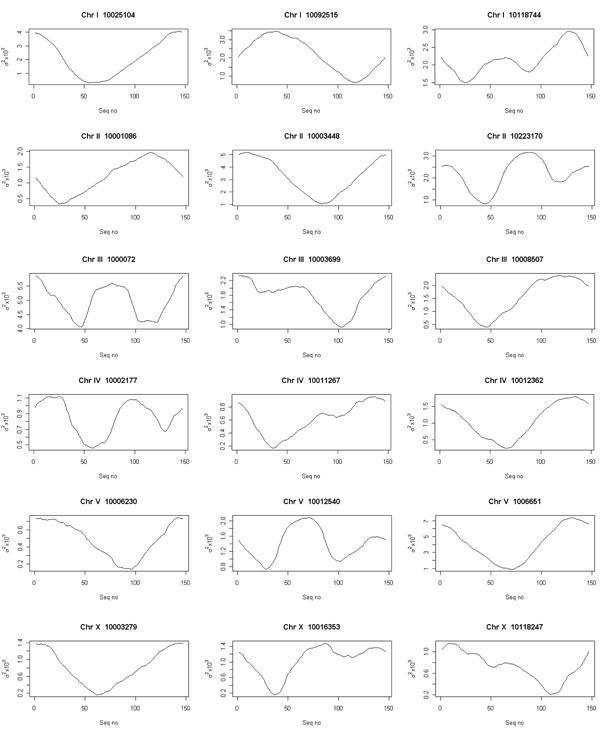
**Curvature dispersion** Curvature dispersion σ^2^ associated with* C. elegans* nucleosome calculated by cyclically permuting the sequences using the theoretical model proposed by De Santis et al [46]. The minima correspond to the molecular bend locus of the fragment.

**Figure 2:**
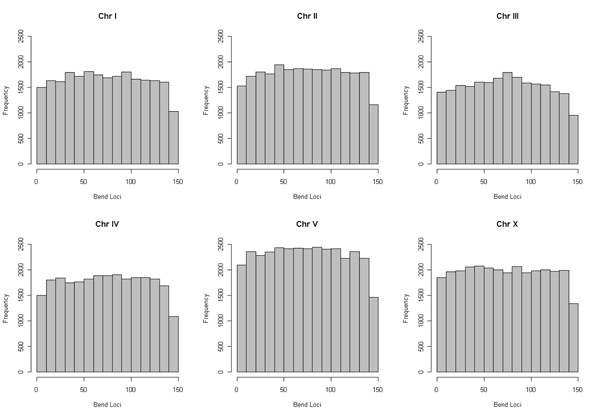
**Histogram of the distribution of the bend loci** Histogram of the distribution of the bend loci associated with* C. elegans* nucleosome as obtained using the theoretical model.

**Figure 3:**
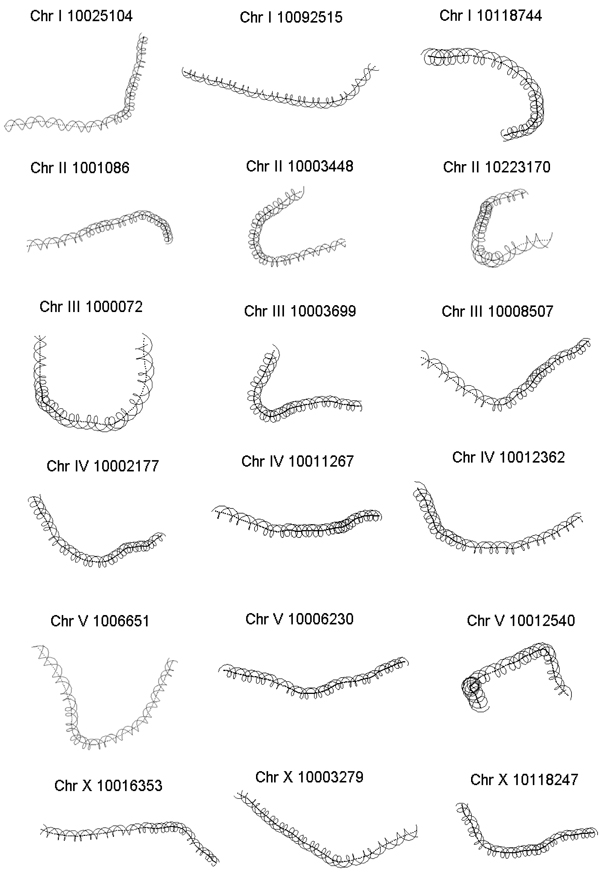
DNA path calculated for the nucleosome DNA sequences represented in Figure 1

The curvature dispersion calculated as the second moment of the curvature vector by cyclically permuting the sequences revealed the molecular bend locus of the nucleosomal DNA sequence. In the interest of brevity, curvature dispersion for three sequences from each of the chromosomes is represented in Figure 1. Curvature dispersion calculations were done for all the sequences in the database. Curvature dispersion retains all the characteristics of the curvature profile, but has the added advantage that it improves the signal to noise ratio. Since curvature dispersion is linearly correlated with gel electrophoretic retardation, calculating curvature dispersion by cyclic permutation of the sequence is equivalent to performing a cyclic permutation assay theoretically [39,49]. The minima of the curve corresponds the bend locus of the fragment. This is equivalent to the experimental cyclic permutation assay in which a linear faster reptating fragment is obtained if its bend locus is destroyed by restriction digestion [45]. Delineating the bend loci associated with the nucleosome DNA sequence helps understand the regions where the curvature is concentrated which in turn helps describe the wrapping of the DNA. Figure 2 shows the distribution of the bend locus for each chromosome as obtained using the theoretical permutation assay. The graphs correspond to the distribution of the minima. Results show a rather even distribution of the loci with relatively fewer loci concentrated at position 140 and beyond. The distribution points to the fact that that nucleosome core region has flexible regions throughout the entire stretch, depending on how it is being packaged. While the histograms in Figure 2 correspond to the minima, it is noteworthy to point out that several nucleosome core sequences had local minimas. Further, the degree of curvature associated with the sequences was also variable. Presence of these local minima reveals a much more complicated deviation of the DNA axis associated with the nucleosome DNA. With a view to understand the deviation of the DNA axis, the DNA paths of the nucleosome core DNA sequences were computed. In the interest of brevity, DNA paths for only three of the nucleosome core sequence for each of the chromosomes is shown in Figure 3. The paths reveal the complex trajectories assumed by nucleosome DNA. It is important to point out that these are theoretically computed results using well accepted models that have been experimentally tested on other systems.

**Figure 4:**
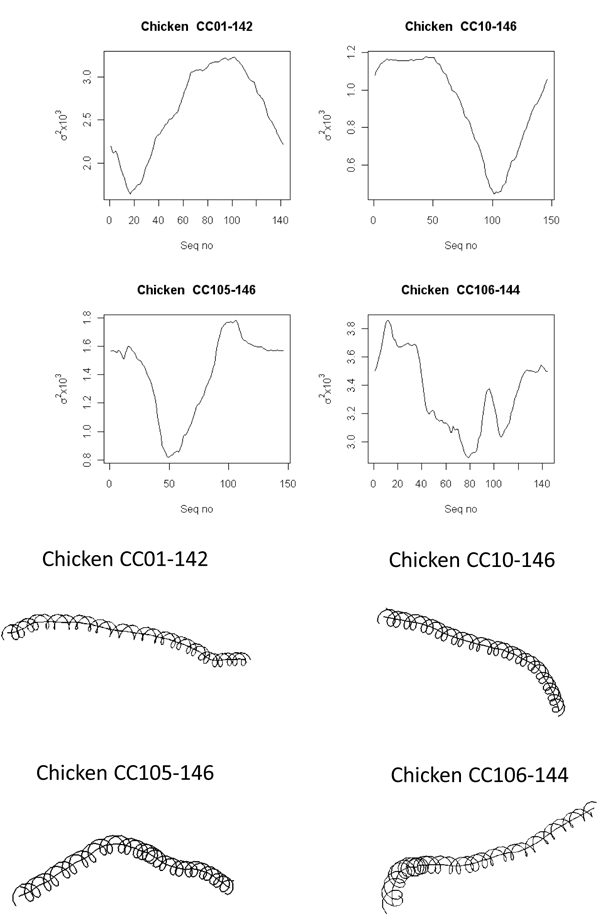
**The upper panel depicts the curvature dispersion** The upper panel depicts the curvature dispersion σ^2^ associated with chicken nucleosome calculated by cyclically permuting the sequences using the theoretical model proposed by De Santis et al [46]. The lower panel is the DNA path calculated for those sequences.

**Figure 5:**
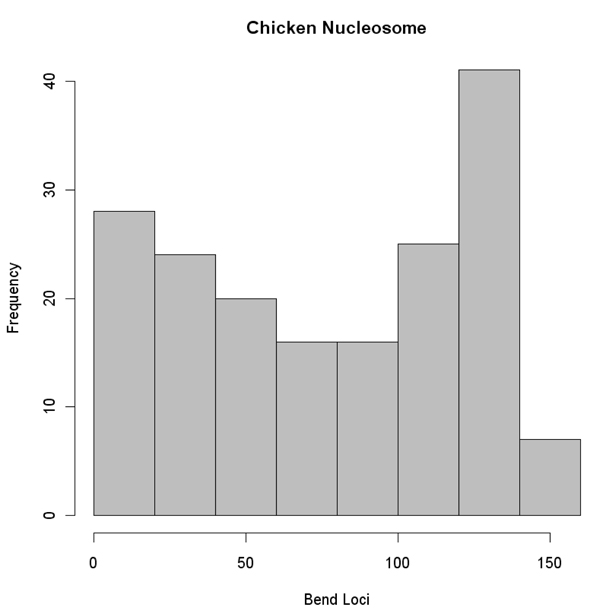
Histogram of the distribution of the bend loci associated with chicken nucleosome as obtained using the theoretical model.

The analysis of the nucleosome DNA sequences from chicken compiled in a pioneering study by Satchwell* et al. *[23] also showed ~10 bp periodicity with respect to AA/TT/TA [14]. In an effort to understand the overall structure of free nucleosome DNA from chicken they were subject to a similar analysis as discussed above in the case of* C. elegans* nucleosome DNA. The results of the analysis are presented in Figure 4. In the interest of brevity data for only 4 out of 177 sequences is shown. The distribution of the molecular bend loci associated with all 177 sequences as obtained using the theoretical model is shown in Figure 5. Even in this case we see that despite the periodicity of certain dinucleotides, the DNA fragments display varying degrees of curvatures and have a broad distribution of their bend loci.

In trying to understand the sequence directed curvatures associated with the nucleosomal DNA, it is important to recap the well established fact that DNA is anisotropic. The anisotropy may be a result of the helical structure of the DNA itself or it may be function of its sequence [54]. In either case it makes it more bendable towards the groove even for unperturbed DNA [55,56]. For the nucleosomal DNA to conveniently wrap around the histone octamer, the sequence repeats should be such that it facilitates this process. Every dinucleotide is capable of deflecting the DNA axis depending on the wedge angles associated with it [43,47]. Periodicity in the distribution of any particular dinucleotide will produce deflection in the DNA axis that will be additive over their individual wedge angle contributions. Towards describing the nucleosome sequence patterns, with the two major competing schools of thought, the "counter- phase" school that claims the RR and YY dinucleotides are distributed in alternating RR/TT fashion and the "in-phase" school that claims the RR and YY dinucleotides are in the same phase within the repeat unit, it is important to understand how these repeats translate into structure and to decipher other messages that nucleosome DNA carry. Further, there are other components that should not be ignored, which include the histone induced bending component and the role of polarization interactions in the wrapping/unwrapping of nucleosomal DNA [57]. The results presented here lend credence to the recent report by Gabdank *et al.*[53], wherein they infer from their analysis that bendability is not the sole reason for positional preference of dinucleotides. The results of this analysis demonstrate flexibility and curvature of nucleosome DNA and reveal that nucleosome DNAs do not conform to the same exact sequence dependent structure.  The nucleosome DNAs have varying degrees of intrinsic curvature, and have bend loci localized at different positions along the sequence.

## Conclusions

Understanding the detailed location of nucleosomes along the DNA is vital to understanding regulation, since positioning of nucleosomes can inhibit or facilitate gene expression [14,58]. With the growing evidence that points to gene regulation at chromatin level, there is an increasing need in defining the sequence structure involved in nucleosome formation. Base pair stacking in nucleosome DNA and bendability sequence pattern recently investigated by Trifonov [18] has underlined the sequence CCGGRAATTYCCGG as the theoretically predicted common pattern of DNA bendability in the nucleosome. While it is important to understand the signal in terms of sequence pattern, only by knowing how that pattern induces deflection in the DNA molecule can one understand the packaging of DNA around the core histones. Further, the nucleosome DNA has been attributed to carrying more messages than just the chromatin code, and is considered the most degenerate code [22,59,60]. From the biological functional perspective, the non-optimal positions of the dinucleotides may actually be an advantage, facilitating important biological processes of replication and transcription. Nature has optimized the chromatin code for multiple functions, making it one of the most difficult feature extraction problems.

## Competing interests

The author has no competing interests associated with the publication of the article.

## Author's contributions

TMN conceptualized the problem, performed all the analysis and wrote the manuscript.
